# Reminiscences of the Insane

**Published:** 1887-02-12

**Authors:** 


### Reminiscences of the Insane.

By a Mental Physician.

(*Continued from page* 254.)

### A Continual Trouble and Anxiety from Childhood to Manhood.

*The Morally Insane.*

Of all the cases which try the patience of, and involve a serious responsibility for, the Mental Physician, none are more perplexing and painful than those in which the intellectual faculties—memory, the capacity for learning and reasoning—are not obviously disordered, but the moral sense is paralysed or perverted. Some of these are a continual source of trouble, anxiety, and grief in childhood and youth as well as in manhood. Others, in consequence of an illness, a moral shock, or a physical injury, undergo a complete transformation of character, and instead of being gentle and considerate, they become injurious to all around them, the plague of the household, and a very dangerous element in family life. It is just here, where medical men recognise the signs of brain disease, that the lawyer can only perceive proofs of wickedness and crime; and the divine, the depravity of heart to which it is his special duty to minister. The schoolmaster also, if he has the misfortune to have such a subject in his school, will be sorely tempted to inflict severe punishment upon him. But neither divine nor schoolmaster succeeds in changing the character.

*Case of a Youth at School.*

A young man in ——— Asylum was an excellent illustration of the class of patients, morally insane from their earliest years. As a child he was dangerous to his brothers and sisters. He was sent to school, and, in fact, to several in succession. One of his schoolfellows thus writes of him: "The boys thought him very clever at his lessons; he would learn them in a shorter time than others did, and then get up to play, and when told to go on, would challenge the master to try if he did not know them. When he was once angry there was no controlling him, and when put into his bedroom he would yell so as to be heard all over the house." At another school his conduct was similar, and his fellow-pupil writes: "Here the same conduct characterised him, and in addition to this, he manifested many acts of violence. Thus, one day when out walking, the master told him to walk behind, in consequence of some misconduct, when the lad attacked him with his fists, and severely hurt him. The master had to knock him down in self-defence. At other times he thought nothing of whipping out his knife and threatening any boy who offended him; and in the night he was fond of standing over a boy in bed with the knife, and threatening his life. The boys teased him, and he, in consequence of this, would for hours fasten himself up in an outhouse while they were at play. At night the boys would fasten his foot to the bed to prevent his attacks upon them." This youth went to sea, and we have before us the journal which he kept on board ship. From this diary a few paragraphs may be extracted, as illustrations of the continual trouble he brought upon himself, and the rough treatment which, although wholly unjustifiable, was, no doubt, directed to what seemed like persistent disobedience. Moral insanity meets with very rough discipline on board ship. It may be well for some of our judges to know that it failed, in the present instance, to do any good.

At Sea.

"We sailed down the Channel with a strong head wind and squalls. The first time we took in sail, the 'earrings' were hauled out upon the main top-sail yard before I had gained the deck. I saw the mate coming for me, and took to the larboard door for an exit, unobserved, but he was too sharp, and nailed me on the galley threshold. Here the villain leathered me with the royal halliards, and sent me up the main rigging. I stood on the top, roaring in concert with the winds, till the second mate sang out to me to hold my tongue and come down. This I did, and was on the point of going on my knees and blessing him for his kindness, when he gave me a crack that sent me backwards, and told me not to get him into a row, and set me on the booby-hatch until the mate's watch was called, when he hurried me up to the top again, as if he did not want the mate to know, though I afterwards heard that it was the mate who told him to call me down. When the mate found I was in the top rigging he sang out for me, and made me go down on my knees and ask his pardon (God forgive me for it), when he kicked me over and told me to go below. After this, the unconquerable pride that possessed me got me upon bad terms with all my shipmates, and the rows between me and the rest of the men frightened the very sharks from the bows. One Sunday afternoon I made free with a piece of pease-pudding which was in the galley. Being caught in the act, I was taken to the mate, who threw a rope round my neck and set the old cook (the prosecutor) to leather me; but it being to his interest to soften the strokes, the skipper came forward and cut about half-a-pound of meat out of my arms. 'You coward,' said I, when he had done, 'I'll tell the owner of you.' This seemed to paralyse him, for he walked away, though any other captain would have ended me for it. One day the mate, having, as he thought, been insulted by one of the men, took me aside and asked me to go into the forecastle and get the aforesaid man to strike me, and then I was to complain about it. I saw the drift of this, but happening to be myself upon ill terms with the man, I was glad enough to be revenged upon him. No sooner said than done. I pushed forcibly against him, upon which he struck me, and upon my complaining to the mate, he knocked the man down and beat him till he was covered with blood. Coming home, I had a row with the second mate. An order had been given to take in the main-royal, which I, not hearing, had gone on to the forecastle, when he came after me, and calling me a dodger, pushed me off the forecastle on to the main deck. I got up and told him that I was no more a dodger than he was; upon which he followed me to the main mast, striking at me at intervals. I then turned round and struck him in the face, which was something new, and all hands were soon mustered on the quarter-deck. We fought round the aft deck hatch, and finally he pushed me down on to the main sheet. I sat down of my own accord, but upon his pushing me over, I seized hold of the back of his neck and kept him down while all the seas in the ocean were coming over him. The mate, who had not been able to eat his breakfast for laughing, came out and told me I had hammered him well; but not so the captain, who looked grave, and sent for me to come aft. He lashed one of my hands to the pump-brake, and the other to a belaying-pin, and he gave me two dozen on my bare back. He had scarcely given the twenty-fourth blow, when crash went the fore-top mast, and gave us three days' job to repair it."

Journal.

Flogged.

On Bad Terms with All.

Flogged Again.

A Fight.

Another Flogging.

The foregoing are merely samples of the kind of life which this morally insane youth passed on board the ship; his hand against every man, and every man's hand against him. When on land, the captain discharged him. He enters in his journal, "My sad propensity to argue soon caused me to fall out with the people of the house where I was. The landlady, being angry at something I said, raised the poker, and gave me such another box with it as Essex got from his royal mistress. There was this difference, however, that I was looking at her full in the face at this time, and instead of laying my hand on my sword, I fingered the tongs, and gave her a clip that brought the blood trickling out of every individual hair of her head."

Assault on Landlady.

It does not, of course, follow from this narrative that the youth was insane. It is only by studying his whole career previously as a child, and subsequently when he was placed in a lunatic asylum, where we closely observed him for several years, that we are justified in pronouncing a definite opinion as to his mental condition.

